# Numerical Simulation Study of Mixed Particle Size Calcination Processes in the Calcination Zone of a Parallel Flow Regenerative Lime Kiln

**DOI:** 10.3390/ma15134609

**Published:** 2022-06-30

**Authors:** Shaopei Duan, Baokuan Li, Wenjie Rong

**Affiliations:** 1 School of Metallurgy, Northeastern University, Shenyang 110819, China; duanshaopei_neu@163.com (S.D.); rongwenjie@smm.neu.edu.cn (W.R.); 2 Key Laboratory of Data Analytics and Optimization for Smart Industry, Ministry of Education, Northeastern University, Shenyang 110819, China

**Keywords:** PFR lime kiln, calcination zone, mixed particle size, numerical simulation, lime calcination

## Abstract

Limestone of different particle sizes is often calcined together to improve production efficiency, but the calcination effect of mixed particle size limestone is difficult to guarantee. To investigate the effect of different particle size combinations on calcination, this study uses a porous media model and a shrinking core model to simulate the calcination process for a single particle size and two mixed particle sizes in a Parallel Flow Regenerative lime kiln (PFR lime kiln). The results of the study show that an increase in void fraction has a small effect on the gas temperature. The temperature also does not change with particle sizes. At the same time, the decomposition is poor near the wall and better the closer to the center of the calcination zone. In addition, when the particle sizes differ by 2 times, the decomposition of small limestone particles had less influence, and the decomposition of large particles was also better. When the particle sizes differ by 3 times, the decomposition of both limestone sizes is more affected, especially for the larger limestone size, where only the outer surface is involved in the decomposition.

## 1. Introduction

The major constituent of quicklime is calcium oxide (CaO), which is produced in industrial production by calcining limestone, whose main component is calcium carbonate (CaCO_3_), at high temperatures to cause a decomposition reaction. Quicklime is widely used in metallurgy, construction, food, agriculture, and other fields, especially in the iron and steel smelting process, which has the highest demand for quantity, as well as high requirements for its chemical activity. The PFR lime kiln is widely used due to its support of multiple fuels and particle sizes, high thermal energy utilization, high output, and excellent chemical activity of the product. Generally, natural gas, with methane (CH_4_) as the main component, is used as fuel gas in the PFR lime kiln, but due to the increasing restrictions on greenhouse gas emissions and the rising price of natural gas in recent years, blast furnace gas (BFG), one of the by-products of steel smelting, is gradually being used as fuel gas in the PFR lime kiln.

The PFR lime kiln is one of the lime shaft kilns. There are many classical studies on lime shaft kilns.

Senegačnik presents that the air excess ratio can be reduced to its optimal level by recirculation of recuperator waste gas [1]. Novel aspects addressed are the simultaneous effects of inner particle heat-conduction and pore-diffusion of the gaseous product of the calcination reaction (CO_2_) modeled by a shrinking core approach [2]. George aims at improving fuel and limestone utilization and reducing waste production in the plant [3]. A new way to evaluate the energetic performance of lime shaft kilns is proposed [4]. A shaft kiln (diameter 4 m and height 21 m) for processing lime is investigated [5]. To present a 1D mathematical model to simulate the lime-burning process in normal shaft kilns [6], Gutiérrez et al. analyzed the energy consumption of vertical kilns [7], Piringer classified lime kilns according to their operating principle and process, and Krause used these classifications to study the calcination process in a PFR lime kiln using CFD-DEM principles [8,9]. Mohammadpour started to use a porous medium model(PMM) to study the gas flow in a lime shaft kiln [10]. Hallaka couple a DPM based on the SCM by comprising differential equations in a single shaft kiln [11], then Duan established a combined porous medium model and shrinking core model to investigate the gas-solid heat transfer and limestone decomposition process of an annular shaft lime kiln and a PFR lime kiln [12,13]. Kashyap analyzes the performance of a regenerative evaporative cooler with all the possible configurations of the air-flow direction by keeping the water flow in a natural (gravity-driven) downward direction [14]. Garcia-Tenorio presents a methodology to select the appropriate signals to produce data-driven models of the kiln as a linear interacting system [15]. Joel Orre developed an OpenModelica model describing the calcination process of limestone in the lime kiln of the SSAB Raahe lime kilns in order to help in the formulation of operation strategies and to choose important parameters to measure and monitor [16].

The latest advances are summarized in the field of modeling packed beds with particle-resolved CFD, i.e., a geometric resolution of every pellet in the bed [17]. Resolved CFD-DEM model is proposed to model the seepage-induced fine particle migration within the gap-graded soils consisting of fine and coarse particles [18]. In the CFD-DEM simulations of fluid-particle systems on sub-particle scale mesh, a smoothed void fraction method (SVFM) is developed to compute the void fraction field based on the particle position and volume [19]. Aimed at optimizing the resin-molding process, a method for numerically analyzing the aggregation and dispersion behavior of the filler in resin composite was proposed [20]. Controlling the size of fragrance microcapsules using designed agitator paddles was investigated and studied with CFD simulation by Hongbin Zhao [21]. Nagata et.al. show that the DEM-CFD simulation could contribute to an appropriate rotor design for uniform dispersion [22]. Ghaffari examines a formulation for the laminar burning velocity that takes into account the effect of particle size using the particle-size-dependent Damkohler and Thiele dimensionless numbers [23].

In studies related to limestone calcination, numerical simulations and experiments are often carried out using homogeneous particle sizes, often increasing or decreasing the overall particle size for the influence of particle size on the calcination process in lime kilns, without considering the degree of influence on the decomposition of limestone when mixed particle sizes are calcined simultaneously. In production, it is more realistic to mix different sizes of lump limestone to participate in calcination. Therefore, this study allows for the use of a reasonable range of mixed-size lump limestone for calcination in future production to achieve better production efficiency while meeting quality requirements.

## 2. Physical Model

The PFR lime kiln is 20 m high and is divided into two chambers of the same structure with a cross channel. Each chamber contains a preheating zone, a calcination zone, and a cooling zone.

**Figure 1:**
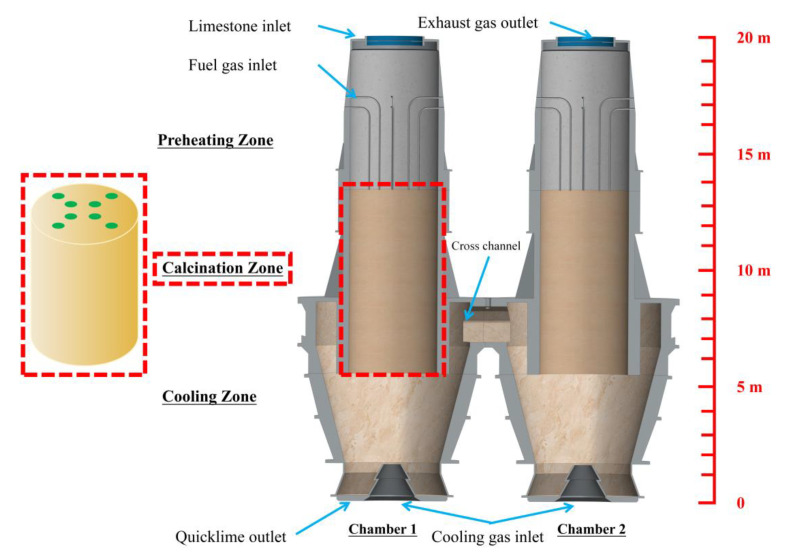
Features of PFR lime kiln composition and the calcination zone.

The PFR lime kiln has a cross channel in the middle and the left and right chambers are identical and symmetrical in structure. The chamber consists of a cooling zone, a calcination zone, and a preheating zone. The preheating zone is 6 m, above the limestone inlet or the exhaust gas outlet, below which there are 8 fuel gas jets, and the lowermost fuel gas inlet is also the boundary between the preheating zone and the calcination zone. The calcination and cooling zones are 9 m and 5 m respectively. The thermal decomposition of limestone mainly takes place in the calcination zone, this paper only focuses on the calcination zone enclosed by the bottom of the fuel nozzle, the interface between the calcination zone and the preheating zone, the interface between the calcination zone and the cooling zone, and the side wall of the kiln chamber (Figure 1).

## 3. Mathematical Models and Boundary Conditions

The production process of lime includes wind resistance and sidewall effect phenomena in flow; convection, radiation, and conduction between solid particles, heat conduction between CaO and CaCO_3_ inside the particles and heat of decomposition of calcium carbonate in heat transfer; and mass transfer process includes decomposition behavior of calcium carbonate and diffusion phenomena of carbon dioxide. Therefore, the interior of the lime kiln is a very complex physicochemical process, and therefore, this study is based on the following hypotheses.

1. Excluding the effect of the external insulation of chambers.
2. Disregard the impurities contained in the limestone particles and treat the particles as pure calcium carbonate.
3. Treating limestone particles as spherical particles.
4. Uniform distribution of limestone particles of different particle sizes within the calcination zone and shift without considering the deflection occurring in the moving process.
5. Gases do not affect the movement of solids.

The simulation uses a quasi-steady state approach to simulate the continuous production.

### 3.1. Mathematical Models

Ergun equation [24]

(1) $$\frac{\left|\nabla p\right|}{L}=\frac{150\mu {\left(1-\gamma \right)}^{2}}{D_{p}^{2}\gamma^{3}}v_{\infty }+\frac{1.75\rho (1-\gamma )}{D_{p}\gamma^{3}}v_{\infty }^{2}$$

(2) $$\frac{1}{\alpha }=\frac{150{\left(1-\gamma \right)}^{2}}{D_{p}^{2}\gamma^{3}}$$

(3) $$C_{2}=\frac{3.5(1-\gamma )}{D_{p}\gamma^{3}}$$

**Figure 2:**
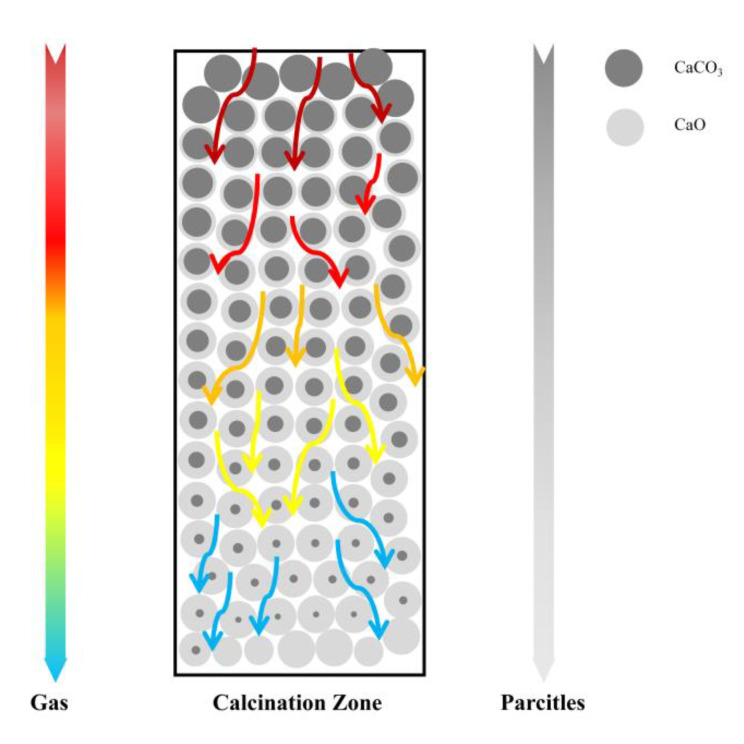
Schematic diagram of gas-solid movement and limestone decomposition process in the calcination zone.

For the decomposition process of individual limestone particles, the shrinking core model can be used for the calculation of the degree of reaction. The shrinking core model (SCM) can be understood as a situation where the outermost layer of calcium carbonate decomposes first when the limestone first starts to react, and as the reaction proceeds, the reaction interface gradually transitions from the outermost layer to the spherical core of the particle. Additionally, the reaction process gradually formed the core of the solid unreacted calcium carbonate, the outer core of the decomposition product’s fluffy calcium oxide situation. The reaction to the end of the unreacted core completely disappears, leaving only the calcium oxide layer, however, the whole limestone particles in the decomposition process, the total volume does not change. As the limestone passes through the calcination zone, it is continuously calcined, and the outer core calcium oxide keeps increasing and the inner core calcium carbonate keeps decreasing. The temperature decreases with height. The schematic diagram of the reaction process in the calcination zone is shown in Figure 2.

The equations of the shrinkage core model are as follows.

(4) $$\frac{\partial r_{{\mathrm{CaCO}}_{3}}}{\partial t}=-k\cdot \frac{M_{{\mathrm{CaCO}}_{3}}}{\rho_{{\mathrm{CaCO}}_{3}}}\cdot R_{D}$$

(5) $$R_{D}=k_{D}\left(p_{eq}-p_{co_{2}}\right)$$

(6) $$k_{D}=0.0001T_{p}\exp\left(-4026/T_{p}\right)\cdot Y_{T.C}$$

(7) $$p_{\mathrm{eq}}=101325\exp\left[17.74-0.00108T_{i}+0.332\log(T_{i})-22020/T_{i}\right]$$

(8) $$Y_{T.C}=\left\{\begin{array}{cc} \frac{480}{T_{P}-958} & T_{P}>1150K\\ 2.5 & T_{P}\leq 1150K \end{array}\right.$$

(9) $$\lambda =\frac{4\pi \lambda_{1}\lambda_{2}}{\lambda_{1}(\frac{1}{r_{c1}}-\frac{1}{r_{c2.m}})+\lambda_{2}(\frac{1}{r_{c1.m}}-\frac{1}{r_{c1}})}$$

Limestone decomposition rate.

(10) $$X_{S}=1-\frac{r_{c2}^{3}}{r_{c1}^{3}}$$

The temperature equations are as follows.

(11) $$\frac{\partial }{\partial t}\left[\varphi_{{\mathrm{CaCO}}_{3}}\rho_{i}c_{\mathrm{pi}}T_{i}\right]+\nabla \left({\vec{v}}_{\mathrm{down}}\varphi_{{\mathrm{CaCO}}_{3}}\rho_{i}c_{\mathrm{pi}}T_{i}\right)=\lambda \left(1-\gamma \right)\left(T_{o}-T_{i}\right)/V_{s}-k\cdot Q_{D}\Delta H_{R}$$

(12) $$Q_{D}=(1-\gamma )\frac{4\pi r_{c1}^{2}}{V_{p}}\times R_{D}$$

Energy equation of the external CaO.

(13) $$\begin{array}{l} \frac{\partial }{\partial t}(\varphi_{\mathrm{CaO}}\rho_{o}c_{\mathrm{po}}T_{o})+\nabla \cdot ({\vec{v}}_{down}\varphi_{\mathrm{CaO}}\rho_{o}c_{\mathrm{po}}T_{o})=\\ \nabla \cdot \left(\left(k_{\mathrm{CaO}}+e_{b}\right)\nabla T_{o}\right)\;+a_{v}h_{v}\left(T_{g}-T_{o}\right)-\lambda \left(1-\gamma \right)\left(T_{o}-T_{i}\right)/V_{s} \end{array}$$

The main reference for thermal conductivity between solids in porous media models is the following Equation [25]

(14) $$e_{b}=16\sigma T_{o}^{3}/\left(3\beta \right)$$

The convective heat transfer coefficient is the following Equation [26]

(15) $$h_{v}=\frac{Nu\cdot \lambda_{g}}{l_{z}}$$

(16) $$Nu=Pr_{}^{1/3}\cdot \frac{1.6274Re_{}^{-0.575}}{\gamma }$$

(17) $$l_{z}=0.0178\rho^{0.596}$$

(18) $$Re=\frac{\rho_{g}\cdot D_{p}\cdot u}{\mu }$$

(19) Pr=ν/a

(20) $$a=\frac{\lambda_{g}}{\rho_{g}c_{\mathrm{pg}}}$$

(21) $$a_{v}=(1-\gamma )\times \frac{S_{p}}{V_{p}}$$

The gas energy equation.

(22) $$\frac{\partial }{\partial t}\left(\gamma \rho_{g}c_{pg}T_{g}\right)+\nabla \left(\gamma \;\vec{v}\rho_{g}c_{pg}T_{g}\right)=\nabla \cdot \left(k_{g}\nabla T_{g}\right)+a_{v}h_{v}\left(T_{o}-T_{g}\right)$$

(23) $$\varepsilon =\left(1-\frac{M_{\mathrm{CaO}}/\rho_{\mathrm{CaO}}}{M_{CaCO_{3}}/\rho_{CaCO_{3}}}\right)\times X_{S}$$

(24) $$\varphi_{g}=(1-\gamma )\cdot \varepsilon +\gamma$$

(25) $$\varphi_{CaCO_{3}}=(1-\gamma )\times (1-X_{S})$$

(26) $$\varphi_{CaO}=1-\varphi_{g}-\varphi_{CaCO_{3}}$$

**Figure 3:**
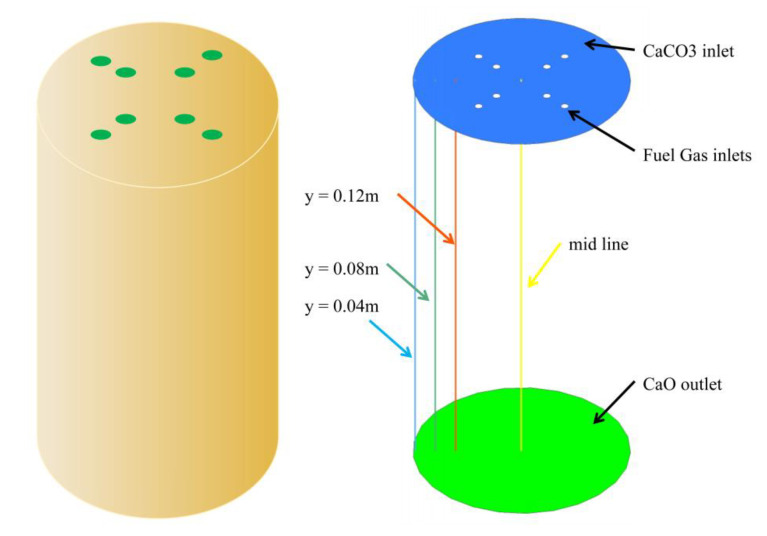
Schematic diagram of calculation area and analysis locations.

In order to facilitate the calculation and analysis, 40 mm limestone particle size was taken as the minimum scale, and the distance from the chamber side wall y = 0.04 m, 0.08 m, 0.12 m, and midline were selected to analyze the variation of gas and solids temperature fields along the longitudinal direction, and the lowermost part of the calcination zone was used as the solids outlet after calcination to analyze the limestone decomposition, as shown in Figure 3.

### 3.2. Boundary Conditions

The inner wall of the chamber is considered as the adiabatic wall and the whole interior of the chamber is considered as a porous media area with CaCO_3_. The fuel gas temperature is taken directly as the boundary condition. The limestone particles in the calcination zone are uniformly distributed and there is no segregation. Other calculation conditions are shown in Table 1.

The equations above are solved using the User Defined Functions (UDF) in ANSYS Fluent. The semi-implicit method (SIMPLE algorithm) was used to solve the pressure and the velocity coupled problem; the turbulence equations were solved in the first-order windward difference format, and the other equations were treated by the second-order windward difference; the convergence factor are set to 10^−6^.

## 4. Results and Analysis

### 4.1. Gas-Solid Temperature Field and Decomposition of 40 mm Uniform Particle Size Limestone

**Figure 4:**
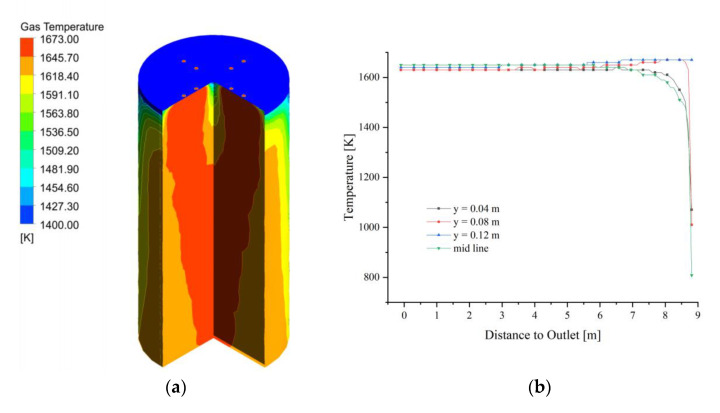
Gas temperature field at 40 mm. (**a**) Gas temperature; (**b**) Gas temperature changes in y = 0.04, 0.08, 0.12 m and mid line.

The fuel gas enters the calcination zone and fills the calcination zone, the lowest temperature is around 1400 K and is concentrated on the position where the limestone has just entered the calcination zone. The temperature in the near-wall area is significantly lower than that in the center of the calcination zone and decreases as it moves down the calcination zone (Figure 4a). In the top area of the calcination zone, the gas temperature distribution is not yet uniform at each position, and the temperature at the position near the fuel gas inlet is more than 150 K higher than in the other positions. And the high-temperature gas from the inlet into the calcination zone after 2 m distance, the temperature drops sharply, especially from the chamber wall 0.08 m and 0.12 m gas drop more. While the calcining area is centered and closer to the chamber wall, the temperature drop is relatively gentle. After the gas enters the calcination zone at a distance of 2 m, the temperature difference between the longitudinal positions of the calcination zone is smaller, and until the CaO outlet, the trend of gas temperature changes is basically the same (as in Figure 4b).

**Figure 5:**
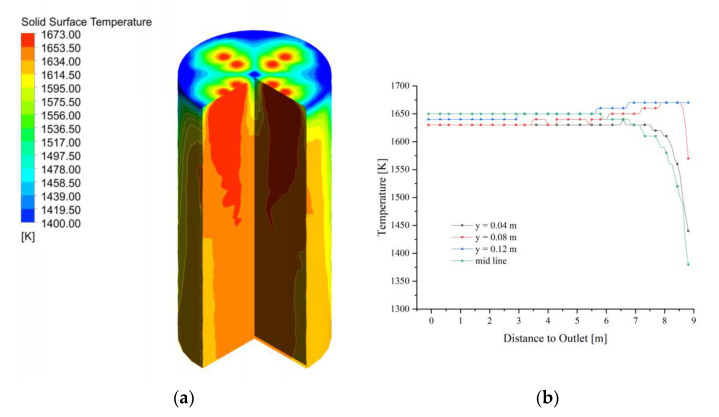
Solid surface temperature field at 40 mm. (**a**) Solid surface temperature; (**b**) Solid surface temperature changes in y = 0.04, 0.08, 0.12 m, and mid line.

Compared to the gas temperature distribution in the calcination zone, the position of the solid surface temperature close to the gas temperature is limited to the upper part of the calcination zone, and the temperature difference among the positions gradually increases as the calcination zone is deepened. At the same time, the temperature gradient on the near-wall side of the calcination zone is also greater than that on the near-wall side of the gas temperature field (Figure 5a). The solid surface temperature increases and decomposition reaction occur as the calcination zone is deepened, while the solid surface temperature of y = 0.04 m and the midline to the height of 8 m in the calcination zone, the temperature has stabilized at about 1620 K, after which no more significant changes occur. Additionally, the solid surface temperature at the position of y = 0.08 m and 0.12 m entered the calcination zone and quickly rise to about 1660 K, which was close to the fuel gas temperature. After that, it decreases slightly and finally stabilizes at about 1650 K as the height of the calcination zone decreases (see Figure 5b).

**Figure 6:**
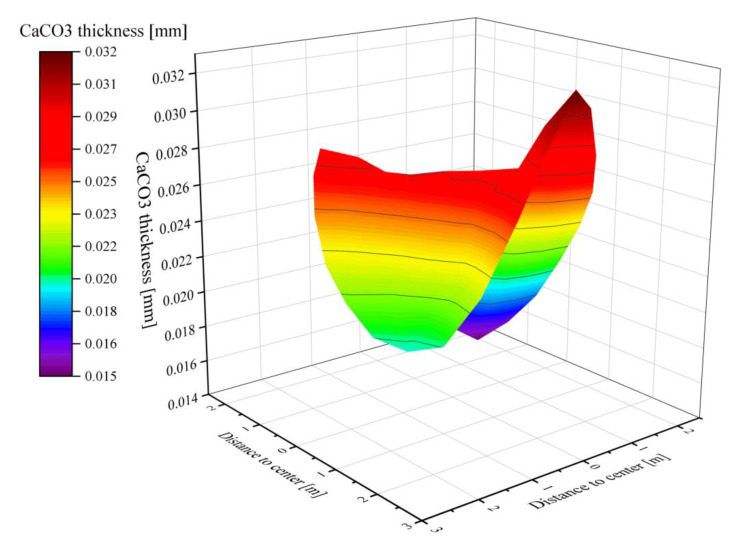
Thickness distribution of CaCO_3_ at the outlet of calcination zone.

When the solids reached the outlet of the calcination zone, most of the calcium carbonate shells finished decomposing into CaO, but there are still CaCO_3_ of different thicknesses in the core. The thickness of CaCO_3_ in some near-wall areas reaches 0.03 mm or more, while the area with the best degree of decomposition has 0.015 mm undecomposed. The undecomposed thicknesses are all very small relative to the 20 mm limestone feed radius and can be considered as fully calcined(Figure 6). However, the thickness of CaCO_3_ at the outlet plane of the calcination zone is not uniform, which is mainly due to the temperature and velocity do not reach the lower part uniformly, which makes the solids in the lower part unevenly heated.

### 4.2. Temperature Field and Decomposition of Limestone with 40 mm and 80 mm

**Figure 7:**
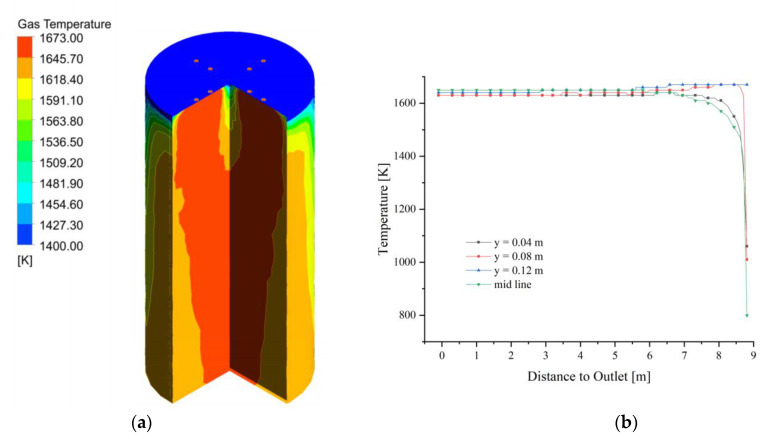
Gas temperature field at 40 mm and 80 mm. (**a**) Gas temperature; (**b**) Gas temperature changes in y = 0.04, 0.08, 0.12 m, and mid line.

After the fuel gas entered the calcination zone filled by 40 mm and 80 mm particles, it quickly filled the upper part, while the center is less affected by the high temperature and shows a larger temperature gradient. As the calcination zone extends downward, the gas temperature gradually decreases, especially the gas temperature near the wall of the chamber decreases more, and the high-temperature area of the gas is concentrated in the central part of the calcination zone (Figure 7a). The gas temperature change in the extension direction of the calcination zone is basically the same as 40 mm, which indicates that the gas diffusion is less affected by the mixed particle size and uniform particle size with different void fractions.

**Figure 8:**
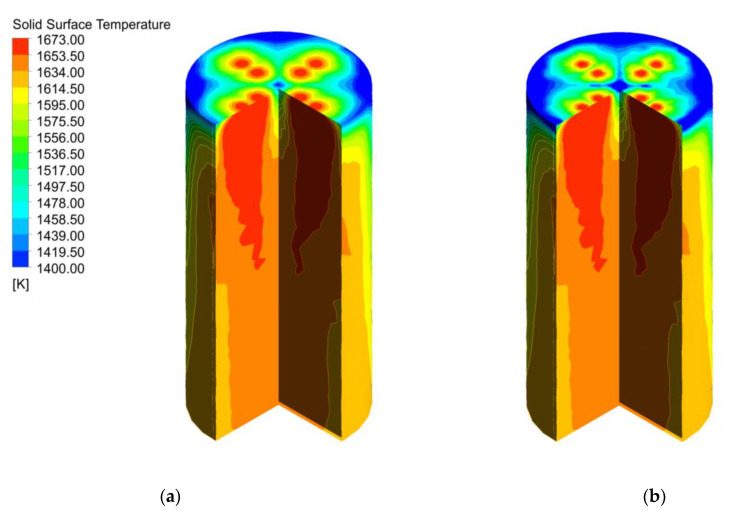
Solid temperature field at 40 mm and 80 mm (**a**) Solid surface temperature of 40 mm; (**b**) Solid surface temperature of 80 mm.

The two particle sizes are uniformly mixed and filled in the calcination zone, the limestone with 40 mm particle size is heated better and its temperature near the wall is higher (Figure 8a), while the limestone with 80 mm particle size takes longer to be heated due to its larger specific surface area, is heated less uniformly, and shows a larger temperature gradient near the wall (Figure 8b).

**Figure 9:**
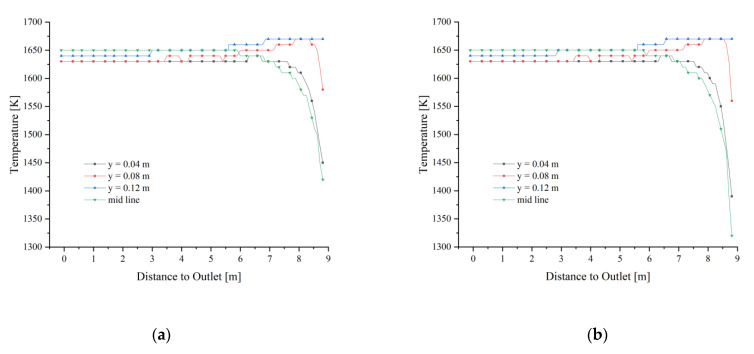
Solid temperature at 40 mm and 80 mm. (**a**) Solid temperature of 40 mm; (**b**) Solid temperature of 80 mm.

When both particle sizes enter the zone, the temperature increase of the small size limestone at y = 0.04 m and the mid line of the calcination zone is more than 100 K greater than that of the large size limestone, while the difference between y = 0.08 m and y = 0.12 m, which is closer to the fuel gas inlet, is only about 30 K. As the calcination zone extends downward, the surface temperatures of the two particle sizes tend to be the same for each distance solid temperature region. (As shown in Figure 9a,b).

**Figure 10:**
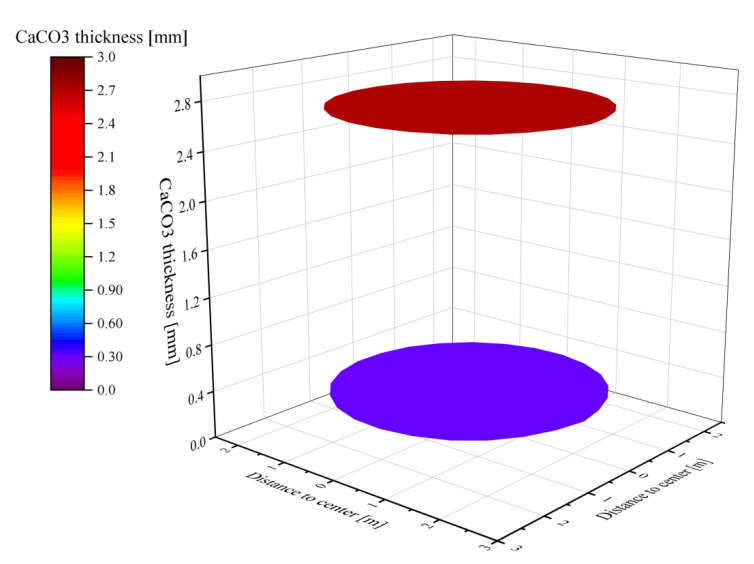
Thickness and distribution of CaCO_3_ of 80 mm particles (**up**) and 40 mm particles (**down**) at the outlet of the calcination zone.

Although the surface temperatures of the two particle sizes are similar, the thickness of CaCO_3_ at the outlet of the calcination zone is still different due to the double difference in particle size. 80 mm particle size CaCO_3_ thickness reaches 2.8 mm, while the thickness of small particle size 40 mm CaCO_3_ is only 0.4 mm. The difference in particle size between the two particles is double, but in terms of CaCO_3_ thickness, large particles are 7 times thickness than small particles (see Figure 10). In contrast, the CaCO_3_ thickness of 40 mm uniform particle size is only 0.015 to 0.03 mm, and the decomposition of limestone of the same size particle in mixed particle size is significantly affected. The main reason is that the reaction time of limestone of larger size is longer, and the heat required is greater, thus reducing the small particle size to be heated and affecting the decomposition of small particle size limestone.

### 4.3. Temperature Field and Decomposition of Limestone with 40 mm and 120 mm

**Figure 11:**
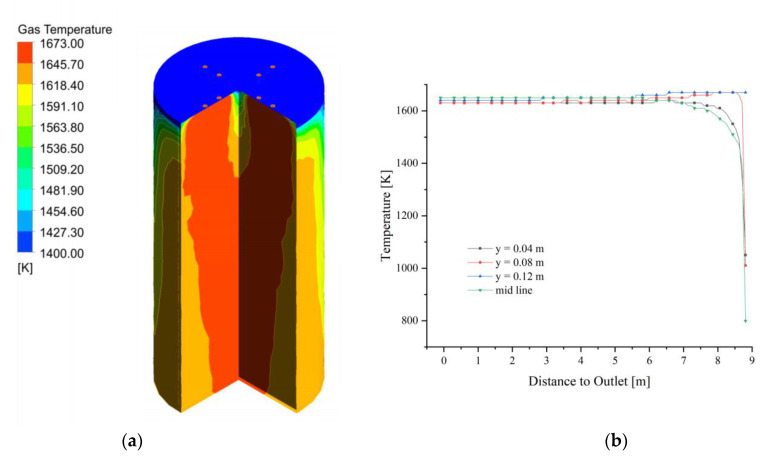
Gas temperature at 40 mm and 120 mm. (**a**) Gas temperature; (**b**) Gas temperature changes in y = 0.04, 0.08, 0.12 m, and mid line.

After the fuel gas enters, the gas temperature field above the calcination zone rises rapidly, and its temperature distribution is basically the same as 40 mm and 80 mm mixed size (as shown in Figure 11a). The high-temperature part of the gas in the calcination zone is still concentrated in the upper part, and with the extension of the calcination zone, the gas temperature change of 40 mm + 120 mm is more similar to that of 40 mm + 80 mm mixed particle size relative to the 40 mm size (as in Figure 11b), which indicates that the gas temperature will not increase with the continuous increase of void fraction even if the void fraction continues to increase.

**Figure 12:**
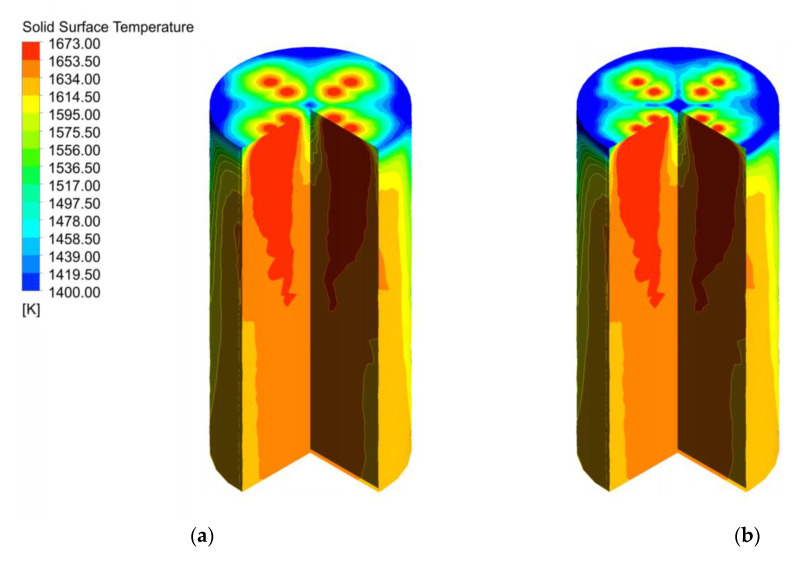
Solid temperature field in the calcination zone at 40 mm and 120 mm. (**a**) Solid surface temperature of 40 mm; (**b**) Solid surface temperature of 120 mm.

The solid surface temperature between 40 mm and 120 mm is shown in Figure 12. 40 mm surface temperature is more uniform, and the temperature near the wall and the center is higher (Figure 12a), compared with 120 mm showing a larger temperature gradient, which is similar to the 80 mm surface temperature under 40 mm + 80 mm in Figure 8.

**Figure 13:**
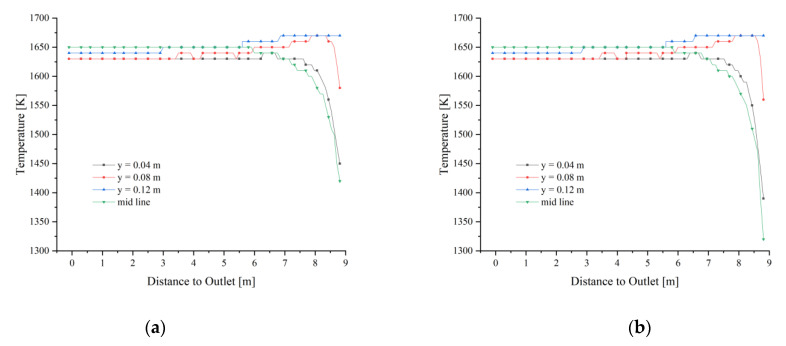
Solid temperature changes in the calcination zone at 40 mm and 120 mm. (**a**) Solid temperature of 40 mm; (**b**) Solid temperature of 120 mm.

The 40 mm particle size limestone heats up faster, and the surface temperature of limestone near the fuel gas inlet at y = 0.08 m and y = 0.12 m quickly reaches more than 1550 K, while the center and near the wall can also reach about 1450 K (Figure 13a). While at 120 mm, the surface temperature at the center is less than 40 mm by about 100 K, while the solid surface temperature near the fuel gas inlet is more similar to that at 40 mm (Figure 13b).

**Figure 14:**
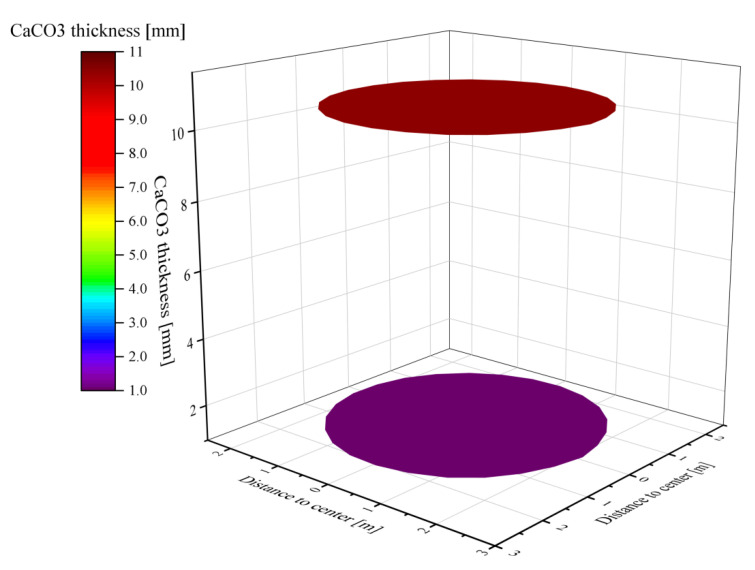
Thickness and distribution of CaCO_3_ of 120 mm particles (**up**) and 40 mm particles (**down**) at the outlet of the calcination zone.

The difference in particle size between the two mixed particle sizes of limestone particles is 3 times, while the thickness of CaCO_3_ under 40 mm is around 1 mm, while the thickness of CaCO_3_ under 120 mm particle size reaches more than 10 mm, which is 4 times of the small particle size (Figure 14). The decomposition degree of small particle size is much better than that of large particle size. However, the CaCO_3_ thickness at 40 mm particle size still exceeds that at 40 mm + 80 mm mixed particle size and is much larger than the remaining CaCO_3_ thickness at 40 mm homogeneous particle size calcination. This indicates that in mixed size calcination, the CaCO_3_ thickness increases with the increase of particle size difference after calcination of small particle size.

**Figure 15:**
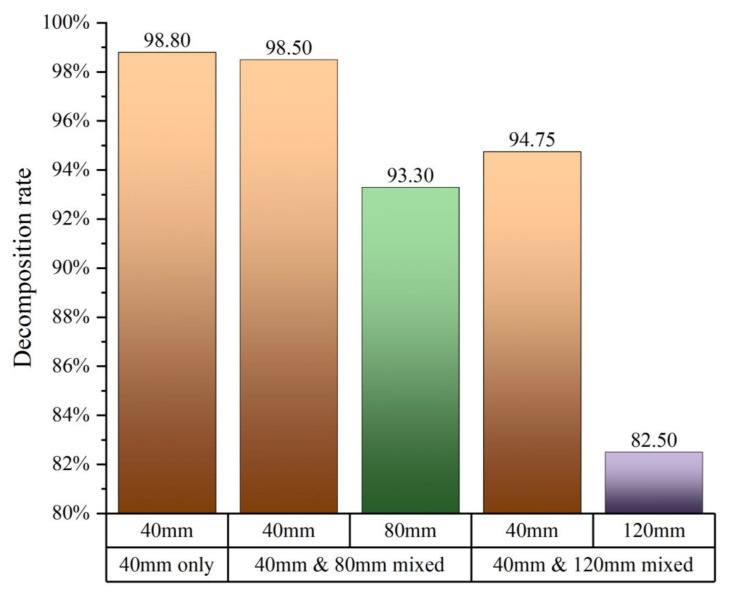
Comparison of decomposition rate under 40 mm uniform particle size, 40 + 80 mm mixed particle size, and 40 + 120 mm mixed particle size.

When mixed size calcination, not only the decomposition of large size limestone is incomplete, but also the heat of fuel gas consumed by it affects the decomposition of small size limestone. The decomposition rate of 40 mm uniform particle size calcination reached 98.8% when the quotient of undecomposed thickness and limestone particle radius is used as the decomposition rate, and the decomposition rate of 40 mm particle size decreases slightly to 98.5% when 80 mm limestone is added to the mixture. When mixing with 120 mm, the decomposition rate of 40 mm is only 94.75%. This indicates that the mixed particle size has a negative effect on the decomposition of small size limestone. It also intensifies with the increase in particle size difference (Figure 15).

## 5. Conclusions

In this study, comparative simulations of the calcination effect of uniform particle and mixed particle are carried out for the calcination zone part in the PFR lime kiln, and the conclusions are as follows:

(1) With the increasing void fraction, the resistance of the porous media region for the gas remains high, and the high-temperature fuel gas has less influence on the gas temperature field in the calcination zone.
(2) Despite the large difference in specific surface area of limestone with different sizes, the difference in surface temperature under each size is smaller, the temperature is also regionally the same as the calcination zone extends downward.
(3) The gas temperature and solid surface temperature gradually increased at y = 0.04 m, y = 0.08 m, and y = 0.12 m along the extension of the calcination zone and reached stability at about 7 m height. However, the temperature at y = 0.04 m, which is closest to the chamber wall, is always lower than that at y = 0.08 m and y = 0.12 m. The reason for this is, firstly, the distance from the fuel gas inlet, which is slightly less heated. The second reason is that the three locations (0.04 m, 0.08 m, and 0.12 m) are each interspersed with a limestone of 40 mm, and the thermal conductivity of limestone directly affects the temperature on the surface.

The study is limited to the calcination zone and does not consider the possibility that cooling air from below may enter the calcination zone after passing through the cooling zone and affect the flow and temperature fields. In addition, the lumpy limestone contains many impurities and their influence on the calcination effect has not been considered. Furthermore, the calcination time for mixed particle size limestone versus single particle size limestone is uncertain due to the quasi-steady state calculations used in this study. Finally, the double-size mixture is also not entirely realistic, and it would be more relevant to carry out a mixture of three or even more limestone sizes and to study the calcination effect.

At the same time, process studies on the involvement of mixed particle size or low calorific value fuel gases in limestone calcination should be combined with equipment optimization and the parameter comparison to make the results more convincing.

## Figures and Tables

**Table 1:** Calculation conditions.

Calculation Conditions	Value	Unit
Fuel gas inlet velocity	20	m/s
Fuel gas inlet temperature	1673	K
Fuel gas nozzle diameter (8)	70	mm
Cooling air inlet velocity	10	m/s
Cooling air inlet temperature	300	K
Cooling air inlet diameter	1000	mm
Material movement speed	1.54	m/h
Initial temperature of material (limestone)	300	K
Average diameter of material	40, 80, 120	mm
Calcium carbonate density	3310	kg/m^3^
Calcium oxide density	2810	kg/m^3^
Calcium carbonate thermal conductivity	2.26	W/m·k
Calcium oxide thermal conductivity	0.07	W/m·k
Calcium carbonate decomposition temperature	1073	K
Void fraction	0.36, 0.41, 0.46	-

## Nomenclature

*ρ*_g_	Fluid density	*ρ*_i_	Density of calcium carbonate
*D*_p_	Diameter of the particles	*c*_i_	Specific heat capacity of calcium carbonate
*α*	Permeability in porous media	*Q*_D_	Particle reaction rate
*C*_2_	Inertial drag coefficient	*ΔH*_R_	Heat of decomposition of calcium carbonate(183,000 J/mol)
$M_{{\mathrm{CaCO}}_{3}}$	Relative molecular mass of calcium carbonate	*V*_p_	Volume of individual particle
$d_{{\mathrm{CaCO}}_{3}}$	Diameter of the internal core calcium carbonate	φ_CaO_	Volume fraction of calcium oxide
$\rho_{{\mathrm{CaCO}}_{3}}$	Density of the calcium carbonate	*ρ*_o_	Density of calcium oxide
*R*_D_	Limestone decomposition rate	*c*_o_	Specific heat capacity of calcium oxide
*k*_D_	Reaction constants	$a_{v}$	Specific surface area of limestone
$p_{\mathrm{eq}}$	Equilibrium partial pressure of carbon dioxide at the front of the reaction zone	$h_{v}$	heat transfer coefficient of gas solid two phase flow
$p_{{\mathrm{CO}}_{2}}$	CO_2_ partial pressure in the environment	σ	Stephen Boltzmann’s constant; 5.6697 × 10^−8^ W/(K^4^·m^2^)
$T_{p}$	Average particle temperature	*β*	Radiation attenuation coefficient of porous media
$T_{i}$	Temperature of internal core calcium carbonate	*Nu*	Nussel number
$Y_{T.C}$	Reaction rate correction factor	*l*_Z_	Stacked bed feature length
$\lambda_{1}$	Thermal conductivity of internal core calcium carbonate	*Pr*	Prandtl number
$\lambda_{2}$	Thermal conductivity of external core calcium oxide	*Re*	Reynolds number
$r_{c1}$	Calcium carbonate radius	a	Thermal diffusion coefficient
$r_{c1.m}$	1/2 calcium carbonate radius	v	Kinematic viscosity of gas
$r_{c2.m}$	Calcium carbonate radius plus 1/2 calcium oxide layer radius	*μ*	Dynamic viscosity of gas
$\varphi_{{\mathrm{CaCO}}_{3}}$	Volume fraction of calcium carbonate	*u*	Velocity of gas movement
